# Diagnosing Noise-Induced Hearing Loss Sustained During Military Service for Men Aged Between 61 and 80 Years

**DOI:** 10.1177/23312165261466799

**Published:** 2026-07-29

**Authors:** Brian C. J. Moore, Petra von Gablenz, Graham Cox, Judy R. Dubno, Lauren K. Dillard, David Lowe, Hedwig E. Gockel

**Affiliations:** 1 Cambridge Hearing Group, Department of Psychology, 2152 University of Cambridge, Cambridge, UK; 2 597470 Institute of Hearing Technology and Audiology and Cluster of Excellence “Hearing4All.connects’’, Oldenburg, Germany; 3 ENT Department (retired), 6397 Oxford University Hospitals NHS Foundation Trust, Oxford, UK; 4 Department of Otolaryngology–Head and Neck Surgery, 2345 Medical University of South Carolina, Charleston, SC, USA; 5 ENT Department, 156705 James Cook University Hospital, Cleveland, UK; 6 Cambridge Hearing Group, MRC Cognition and Brain Sciences Unit, 2152 University of Cambridge, Cambridge, UK

**Keywords:** noise-induced hearing loss, effects of age, diagnosis, older men, progression of hearing loss

## Abstract

A recently described method (rM-NIHL) for diagnosing noise-induced hearing loss (NIHL) sustained during military service for men aged up to 60 years was developed based on the shapes of the audiograms of noise-exposed military personnel and a control group matched in age but without known noise exposure. Here, the applicability of the rM-NIHL method to men aged 61 to 80 years was evaluated, based on a database including 329 military noise-exposed men (ExpDB) and a database including 123 non-exposed men (ContDB). The method had high sensitivity but poor specificity. For further analysis, ExpDB was randomly split into ExpDB1 and ExpDB2, and ContDB was randomly split into ContDB1 and ContDB2. ExpDB1 and ContDB1 were used for the development of new methods and ExpDB2 and ContDB2 were used for evaluation. Modification of the criteria used in the rM-NIHL method led to a better balance between sensitivity and specificity, but the overall diagnostic performance was only moderate. A new method, denoted M-NIHL60+, was developed based on a weighted average of the age-corrected hearing thresholds across all frequencies from 1 to 8 kHz. This method gave a reasonable balance between sensitivity and specificity and gave higher diagnostic performance than the original rM-NIHL method or the rM-NIHL method with modified criteria. Comparison of data for older and younger exposed groups (assessed previously) supported the hypothesis that the rate of change of thresholds with age for noise-exposed individuals is greater for frequencies where the initial NIHL is small than for frequencies where it is large.

## Introduction

Noise-induced hearing loss (NIHL) sustained during military service (denoted M-NIHL) often involves exposure to very intense impulsive sounds and is associated with a more variable pattern of audiometric thresholds than NIHL produced by exposure to steady broadband sounds, such as may occur in factories (Lowe & Moore, 2021; Moore, 2020; Passchier-Vermeer, 1974). Specifically, sustained exposure to high-level steady broadband noise often results in a notch or bulge in the audiogram centered near 4 kHz (Passchier-Vermeer, 1974), which is often used to diagnose NIHL (Coles et al., 2000; Niskar et al., 2001; Phillips et al., 2010; Pudrith et al., 2022), while M-NIHL is sometimes but not always associated with such a notch or bulge (Lowe & Moore, 2021; Moore, 2020). This paper is concerned with methods of diagnosing M-NIHL for military personnel whose noise exposure occurred mainly during military service. Other diagnostic methods may be applicable for NIHL caused by different types of exposures (Moore et al., 2022a). Note that methods for diagnosing M-NIHL are not intended to distinguish M-NIHL from other types of NIHL.

To make a diagnosis of M-NIHL, plausible causes of hearing loss other than noise exposure during military service, including significant noise exposure from other occupations or leisure activities, should be ruled out as far as possible. This requires a thorough medical history to be obtained for the individual concerned (Moore et al., 2022a). In what follows, it is assumed that this requirement has been satisfied. However, it should be noted that M-NIHL can coexist with other causes of hearing loss.

A method specifically aimed at the diagnosis of M-NIHL was described by Moore (2020). A positive diagnosis for a given ear of a given individual was based on the following requirements:

R0: Evidence of exposure to noise with the potential for producing NIHL.
R1: The hearing threshold level (HTL) at any one of 3, 4, 6, or 8 kHz should be at least 10 dB higher than the HTL at 1 or 2 kHz.
R2a: The presence of a notch or bulge in the audiogram centered at 3, 4 or 6 kHz and exceeding a criterion magnitude, Mag(Notch).
R2b: The hearing loss at high frequencies being greater than would be expected from age alone by a criterion amount, Excess(HF).

For a positive diagnosis to be made, the first two requirements, R0 and R1 must both be met, and either or both of the third and fourth requirements, R2a and R2b, must be met. The method was estimated to have high sensitivity (the proportion of individuals with M-NIHL who receive a positive diagnosis, referred to as “hits”), but poor specificity (the proportion of individuals without M-NIHL who receive a negative diagnosis; it is equal to 1 minus the proportion of false positives) (Moore & von Gablenz, 2021). The method was later modified by adjusting the two criteria to improve the specificity of the method (Moore et al., 2022b) at the cost of a small reduction in sensitivity. For the modified method, called the rM-NIHL method, the criterion value of Mag(Notch) was 14.5 dB and the criterion value of Excess(HF) was 19 dB. For details, see Moore et al. (2022b).

A third method of diagnosing M-NIHL, based on a deep neural network, was developed by Moore and Schlittenlacher (2023) and called the MLP(18) method. The method was trained and evaluated using the same databases as for the rM-NIHL method. The MLP(18) method had slightly higher sensitivity and markedly higher specificity than the rM-NIHL method.

The rM-NIHL and MLP(18) methods were developed using noise-exposed and control populations of males with ages up to 60 years. Machine-learning methods based on specific populations often do not generalise well to other populations, and informal evaluations indicated that the MLP(18) method had poor specificity for men aged over 60 years. Insufficient data were available to assess whether the rM-NIHL method was applicable to women of any age or to men aged over 60 years.

Since the publication of the rM-NIHL method, the authors have gathered the audiograms and ages of reasonably large samples of noise-exposed male military personnel and non-exposed men aged 61 to 80 years. The present paper had three purposes. The first two were: (1) to assess the sensitivity and specificity of the rM-NIHL method and the Coles-Lutman-Buffin (CLB) method (Coles et al., 2000) when applied to data from men aged 61-80 years; (2) if necessary, to modify the criteria used in the rM-NIHL method or to develop new criteria that lead to better diagnostic performance for men aged 61 to 80 years. It was anticipated that the rM-NIHL method would perform more poorly for men aged 61 to 80 years than for men aged up to 60 years, because age-associated hearing loss (AAHL) increases with increasing age, making it more difficult to disentangle the effects of age and of noise exposure (Dillard et al., 2025c; Hederstierna & Rosenhall, 2016).

The third purpose of this paper was to test the hypothesis that exposure to noise during military service can accelerate the subsequent progression of hearing loss with increasing age for frequencies where hearing loss at the end of military service is mild or absent (typically low frequencies), but has no effect or slows the subsequent progression of hearing loss for frequencies where the hearing loss is moderate or severe (typically high frequencies) (Dillard et al., 2025c; Moore, 2021; Moore & Lowe, 2022). For brevity in what follows, this is referred to as the “acceleration/deceleration” hypothesis. For example, according to ISO 7029 (2024), for a non-noise exposed man at the 50^th^ percentile, the HTL would be expected to increase (worsen) by about 5 dB at 8 kHz between the ages of 30 and 40 years. If a man ceases military service at age 30 years, and that man has an HTL of, say, 10 dB HL at 8 kHz at the end of service, then acceleration would be expected; the HTL should increase by more than 5 dB over the next 10 years. On the other hand, if that man has an HTL of, say, 50 dB HL at 8 kHz at the end of service, then deceleration would be expected; the HTL should increase by less than 5 dB over the next 10 years. If this hypothesis is correct, the hearing loss of noise-exposed individuals, relative to AAHL values, should vary less with frequency for men aged 61 to 80 years than for men aged below 60 years.

## Method

### Study Populations

There is no “gold standard” for diagnosing M-NIHL. However, a lower bound to the sensitivity of a diagnostic test can be estimated by applying the test to a population that is highly likely to have M-NIHL. It has been shown that, provided a test has some diagnostic value (the proportion of hits being greater than the proportion of false positives), the sensitivity of the test is equal to or greater than the proportion of individuals receiving a positive diagnosis (Moore & Gockel, 2026). Similarly, specificity can be estimated using a matched population that is unlikely to have had significant noise exposure; the proportion of false positives is equal to or less than the proportion of individuals in the control population who receive a positive diagnosis (Moore & Gockel, 2026).

Here, as in Moore (2020), Lowe and Moore (2021), and Moore et al. (2022b), the audiograms and ages of former male military personnel were used to estimate sensitivity. All had seen active service in the UK military and all were claiming compensation for M-NIHL, although for some the primary complaint was tinnitus and/or hyperacusis. None had a significant history of exposure to ototoxic medications (although some had been exposed to jet fuel and other ototoxic substances), or of current or previous ear diseases, significant head injury, or any relevant family history. None reported exposure to intense sounds other than during military service. There were 339 men in the noise-exposed group. Their average age was 66.3 years (range 61 to 80 years), with a standard deviation (SD) of 4.1 years. The number of years of service ranged from about 4 to 30 years with a mean of about 15 years. Most had ceased service 25 to 48 years before the date when their audiogram was obtained for the current study. The audiograms of this group were all obtained using the methods recommended by the British Society of Audiology (2018).

Specificity estimates were obtained using a control population screened to exclude significant noise exposure from any source. The data for the control population were taken from the German database described by von Gablenz and Holube (2016) and the “Medical University of South Carolina Longitudinal Cohort Study of Age-related Hearing Loss” database described by Dillard et al. (2024; 2025a; 2025b). Audiograms for the former were obtained using the methods recommended in ISO 8253-1 (2010). Audiograms for the latter were obtained using the methods recommended by the American Speech-Language-Hearing Association (2005). These methods are similar to those recommended by the British Society of Audiology (2018).

The control population had no excessive self-reported noise exposure, no self-reported history of ear diseases, and no evidence of conductive hearing loss. There were 123 men in the control group. Their average age was 70.6 years (range 61 to 80 years, SD = 5.9 years). While the control group was slightly older on average than the exposed group, this was not regarded as a serious problem since the diagnostic methods considered here all take AAHL into account. It was assumed that the great majority of the control population did not have NIHL. However, since many of the individuals in the control population were not medically examined, some of them may have had causes of hearing loss other than noise exposure.

Since the HTLs of some of the noise-exposed population were recorded as 100 dB HL when the audiometer could not produce output levels above 100 dB HL, it was decided to set any HTLs above 100 dB HL to 100 dB HL, for both populations. This had little effect on the outcome of the diagnostic methods considered here, since all ears with HTLs of 100 dB were classified as having NIHL.

It was anticipated that the rM-NIHL method might need to be modified to improve its diagnostic performance for men aged 61 to 80 years. To allow a fair evaluation of any modified method, the databases used to develop the modified method were different from those used to evaluate it. To achieve this, the database including 339 noise-exposed men was split into two roughly equivalent databases. This was done in the following way. The order of the men was randomly shuffled, and then the database was split into two, with 165 in the first and 164 in the second, denoted ExpDB1 and ExpDB2. This process was repeated several times until the average ages and the average audiometric thresholds (across the frequencies 1, 2, 3, 4, 6, and 8 kHz and across ears) were similar for ExpDB1 and ExpDB2. The average age was 66.1 years (standard deviation, SD = 4.0 years) for ExpDB1 and 66.4 years (SD = 4.2 years) for ExpDB2. The average audiometric thresholds (1 to 8 kHz) were 49.9 dB HL (SD = 15.3 dB) for ExpDB1 and 50.4 dB HL (SD = 14.4 dB) for ExpDB2. The 123 individuals in the control database were similarly split into two databases, denoted ContDB1 and ContDB2. The average age was 70.5 years (standard deviation, SD = 5.7 years) for ContDB1 and 70.5 years (SD = 6.1 years) for ContDB2. The average audiometric thresholds (1 to 8 kHz) were 37.8 dB HL (SD = 16.8 dB) for ContDB1 and 37.4 dB HL (SD = 15.0 dB) for ContDB2. The databases ExpDB1 and ContDB1 were used to develop a modified version of the rM-NIHL method. The databases ExpDB2 and ContDB2 were used for evaluation of the modified method.

## Results

### Hearing Threshold Levels for Each Group

**Figure 1.**
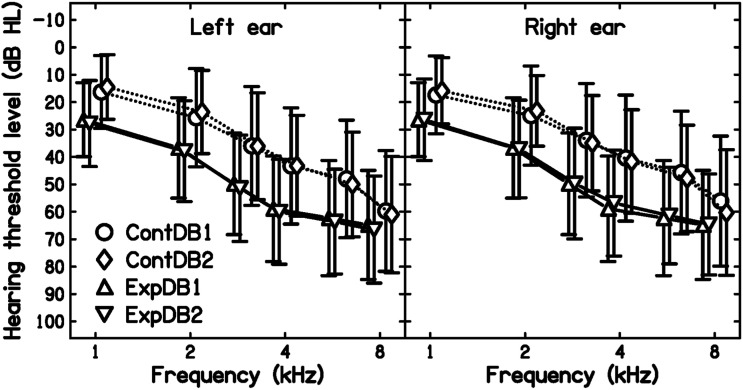
Mean audiometric thresholds for each group for frequencies from 1 to 8 kHz, shown separately for the left and right ears. Error bars show ±1 standard deviation (SD). For visual clarity, the data points are slightly shifted from their correct positions on the abscissa

Means and SDs of the HTLs for each ear of each group for frequencies from 1 to 8 kHz are shown in Figure 1. On average the hearing loss at high frequencies (4, 6 and 8 kHz) for the two exposed groups was greater for the left than for the right ears, as has been reported previously for military personnel (Keim, 1969; Lowe & Moore, 2021; Moore, 2020). This has been attributed to greater noise exposure of the left ear, on average, partly because of the way that a rifle is usually fired from the right shoulder, which results in partial shielding of the right ear via the head-shadow effect (Keim, 1969). However, the average difference across ears found here was markedly smaller than found in earlier studies. For example, Lowe and Moore (2021) found that for noise-exposed former military personnel the average HTL across 4, 6 and 8 kHz was 58.3 dB HL for the left ear and 50.0 dB for the right ear, a difference of 8.3 dB. For the older exposed men evaluated here, the corresponding values were 62.0 dB HL for the left ear and 61.2 dB for the right ear (a difference of 0.8 dB) for ExpDB1 and 63.4 dB HL for the left ear and 61.5 dB for the right ear (a difference of 1.9 dB) for ExpDB2. The small across-ear differences found here for the exposed groups were similar to those for the two control groups: for ContDB1 the average HTL across 4, 6 and 8 kHz was 50.3 dB HL for the left ear and 47.4 dB for the right ear, a difference of 2.9 dB; for ContDB2, the corresponding values were 53.3 dB HL for the left ear, 50.9 dB for the right ear, a difference of 2.4 dB. However, it should be remembered that the control groups were on average about 4 years older than the exposed groups. The small difference between ears found here for the exposed groups suggests that with increasing age, the hearing loss for the less affected ear “catches up” with that for the more affected ear, consistent with the “acceleration/deceleration” hypothesis.

### Hearing Threshold Levels Relative to AAHL Values

**Figure 2.**
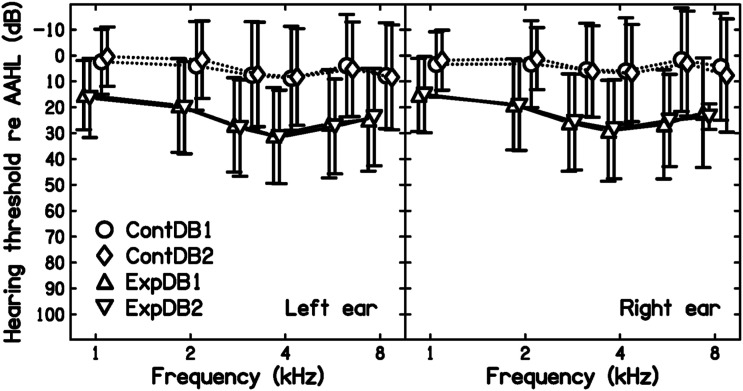
As figure 1 but with HTLs expressed relative to age-expected values taken from ISO 7029 (2017)

Figure 2 shows means and SDs of the HTLs relative to AAHL values taken from ISO 7029 (2024). As expected, these values fall close to 0 dB for the two control groups. The fact that the average values were slightly worse than 0 dB HL may reflect the fact that individuals were not excluded from the control groups if they had self-reported hearing difficulties, while at least some of the samples used to develop ISO 7029 (2024) were screened to exclude such individuals. It is noteworthy that a few individuals in the control groups had HTLs that were much worse than expected from ISO 7029 (2024). For example, one individual in ContDB1, aged 72 years, had HTLs of 100 dB for all frequencies from 2 to 8 kHz, much worse than the AAHL values from ISO 7029 (2024), which are 47, 55, 63, 73 and 81 dB HL at 2, 3, 4, 6, and 8 kHz at the 5^th^ (worst) percentile. Presumably, this individual had some hearing pathology despite having no self-reported history of ear diseases. The age-corrected threshold values (HTLs relative to AAHLs) were similar for the two exposed groups. Both groups showed worse hearing than expected for their age over a wide frequency range.

For the younger noise-exposed group assessed by Lowe and Moore (2021) (mean age 47.1 years, SD = 9.0 years), the mean age-corrected threshold at 4 and 6 kHz was higher than the age-corrected threshold at 1 kHz by 29.6 dB for the left ear and 23.5 dB for the right ear. For the older group tested here, the corresponding differences were 13.3 dB and 12.2 dB, respectively. Assuming that the age-corrected thresholds reflect the amount of NIHL (Moore et al., 2022a), these findings indicate that while NIHL for noise-exposed younger men is markedly greater at high frequencies (4 and 6 kHz) than at l kHz, NIHL for noise-exposed older men varies less across frequency. Again, this is consistent with the “acceleration/deceleration” hypothesis.

### Evaluation of the rM-NIHL and CLB Methods for Older Men

The rM-NIHL method for diagnosing M-NIHL (Moore et al., 2022b) and the CLB method for diagnosing NIHL (Coles et al., 2000) were evaluated using the data for ExpDB1 and ExpDB2 combined and for ContDB1 and ContDB2 combined. For details of these methods, the reader is referred to the original publications. An individual was deemed to have M-NIHL if the diagnosis was positive for either ear.

For the rM-NHL method and the combined data for the exposed groups, the proportion of positive diagnoses was 0.836 for the left ear, 0.805 for the right ear, 0.726 for both ears and 0.915 for either ear. For the combined data for the control groups, the proportion of positive diagnoses (i.e. false positives) was 0.455 for the left ear, 0.407 for the right ear, 0.301 for both ears and 0.561 for either ear. Thus, based on “either ear”, sensitivity was high (0.915) but specificity (0.439) was poor.

The overall quality of a diagnostic test can be quantified using the detectability index, *d'*, which is calculated from the proportion of “hits” (sensitivity) and “false alarms” (1 − specificity):

$$d^{\prime }=z\left(\text{hit}\;\text{rate}\right)–z\left(\text{false}\;\text{alarm}\;\text{rate}\right)$$

where function *z*(*p*), *p* ∈ [0,1], is the inverse of the cumulative Gaussian distribution (Green & Swets, 1974). Based on the hits and false positives for the case of “either ear”, the *d'* value for the rM-NIHL method was 1.31 (95% confidence intervals, CI, based on estimation of the variance of *d*′ using the method of Gourevitch and Galanter (1967), 1.02 to 1.60). For comparison, when assessed using men aged up to 60 years, the rM-NIHL method had a sensitivity of 0.979, a specificity of 0.630 and a *d'* value of 2.37 (CI 1.83 to 2.90) (Moore et al., 2022b). This is consistent with the expectation that the rM-NIHL method would perform more poorly for the older than for the younger men.

For the CLB method, the proportion of positive diagnoses for the combined exposed group was 0.629 for the left ear, 0.660 for the right ear, 0.508 for both ears and 0.781 for either ear. For the combined control group, the proportion of false positives was 0.325 for the left ear, 0.366 for the right ear, 0.211 for both ears and 0.480 for either ear. Based on the hits and false positives for the case of “either ear”, the *d'* value for the CLB method was 0.826 (CI 0.56 to 1.09), lower than the value of 1.31 for the rM-NIHL method (but with slight overlap of the CIs).

### Effect of Varying the Criteria for the rM-NIHL Method

The effect of varying the criteria used in the rM-NIHL method, namely Mag(notch) and Excess(HF) was assessed. The goal was to find the criteria leading to the highest *d'* value, subject to the constraint that the proportion of false positives for the either-ear case should not exceed 0.4. The effect of varying the criteria was initially assessed using ExpDB1 and ContDB1. Many combinations of the values of Mag(Notch) and Mag(ExcessHF) were evaluated. The values that best met the goal were Mag(Notch) = 17.0 dB and Mag(ExcessHF) = 27.0 dB. With these values, based on the “either ear” case, the proportion of positive diagnoses for ExpDB1 was 0.842 and the proportion of false positives for ContDB1 was 0.393.

Table 1 shows values of sensitivity, specificity, and *d'* based on a positive diagnosis for either ear, both ears, and the left and right ears considered separately (with the results then averaged across ears), for the original rM-NIHL method and for the method with revised criteria. Generally, the revised criteria led to lower sensitivity but higher specificity than for the rM-NIHL method, the *d*′ values being similar with the original and modified criteria.

**Table 1.** Rows 2-4 Show Values of Sensitivity, Specificity, and *d'* for the rM-NIHL Method Using ExpDB1 and ContDB1 Based on a Positive Diagnosis for: (1) Either Ear; (2) Both Ears; (3) Each Ear Separately. Rows 4-6 Show Corresponding Values With Revised Criteria for Mag(Notch) and Excess(HF)

Method	​	Sensitivity	Specificity	*d'*
rM-NIHL	Either ear	0.915	0.475	1.31
rM-NIHL	Both ears	0.727	0.738	1.24
rM-NIHL	Each ear separately	0.821	0.607	1.19
Revised criteria	Either ear	0.842	0.607	1.27
Revised criteria	Both ears	0.582	0.902	1.50
Revised criteria	Each ear separately	0.712	0.754	1.25

### Sensitivity and Specificity Values of the Original rM-NIHL Method and the Method With Revised Criteria Using ExpDB2 and ContDB2

Table 2 shows values of sensitivity, specificity, and *d'* for each of the three cases when assessed using ExpDB2 and ContDB2, with the original criterion values of Mag(Notch) and Mag(ExcessHF) and with the revised criterion values. The patterns of results was similar to that for Table 1. The revised criteria led to a better balance between sensitivity and specificity. The *d'* values with the revised criteria were slightly higher than with the original criteria, but the differences were not significant. For the “either ear” case, the lower and upper CIs were 0.71 and 1.55 with the original criteria and 0.89 and 1.68 with the revised criteria.

**Table 2.** As Table 1 but for Results Obtained Using ExpDB2 and ContDB2

Method	​	Sensitivity	Specificity	*d'*
rM-NIHL	Either ear	0.915	0.403	1.13
rM-NIHL	Both ears	0.720	0.661	1.00
rM-NIHL	Each ear separately	0.817	0.532	0.98
Revised criteria	Either ear	0.841	0.613	1.29
Revised criteria	Both ears	0.579	0.887	1.41
Revised criteria	Each ear separately	0.711	0.750	1.23

### A New Diagnostic Method Based on HTLs Relative to AAHL Values at all Frequencies From 1 to 8 kHz

It is apparent from Figure 2 that the noise-exposed groups had higher HTLs relative to AAHL values over a wide frequency range. Therefore, it seemed worthwhile evaluating a diagnostic method, denoted M-NIHL60+, based on a weighted average of the relative HTLs across all frequencies from 1 to 8 kHz. The weighting was intended to reflect the extent to which the HTL at each frequency was informative: this increases with increasing mean difference between exposed and non-exposed groups and decreases with increasing standard deviation of the HTLs at a given frequency. The weighting for each frequency, *f*, was derived from the data for ExpDB1 and ContDB1 as follows:

(1) The relative HTL (measured HTL minus AAHL value) was averaged across ears separately for each group and each frequency.
(2) The difference in average relative HTL between the two groups was calculated; this is denoted Diff(*f*).
(3) The SD of the relative HTLs was averaged across ears and across groups, to give a global measure of variability at frequency *f* denoted SD(*f*).
(4) The weight assigned to frequency *f* was [Diff(*f*)/SD(*f*)]/*K*, where *K* is equal to the sum of the values of [Diff(*f*)/SD(*f*)] across frequency.

The weights for each frequency obtained in this way were 0.171, 0.165, 0.171, 0.188, 0.182 and 0.123, at 1, 2, 3, 4, 6, and 8 kHz. For each ear of each individual, the relative HTLs at each frequency were multiplied by the appropriate weight and summed to give a single value, *R*, in dB. The value of *R* is an overall measure of hearing loss relative to AAHL values. M-NIHL was deemed to be present when *R* exceeded a criterion value. The criterion value was chosen, using ExpDB1 and ContDB1, to give to the highest *d'* value subject to the constraint that the proportion of false positives for the either-ear case should not exceed 0.4. The resulting criterion value was 9.4 dB. The sensitivity and specificity using this criterion value were then evaluated using ExpDB2 and ContDB2. The outcomes are shown in Table 3.

**Table 3.** Rows 2-4 Show Values of Sensitivity, Specificity, and *d'* for the M-NIHL60+ Method Using ExpDB1 and ContDB1 Based on a Positive Diagnosis for: (1) Either Ear; (2) Both Ears; (3) Each Ear Separately. Rows 4-6 Show Corresponding Values for ExpDB2 and ContDB2.

Database	​	Sensitivity	Specificity	*d'*
ExpDB1 and ContDB1	Either ear	0.879	0.623	1.48
ExpDB1 and ContDB1	Both ears	0.758	0.721	1.29
ExpDB1 and ContDB1	Each ear separately	0.818	0.673	1.36
ExpDB2 and ContDB2	Either ear	0.909	0.581	1.54
ExpDB2 and ContDB2	Both ears	0.793	0.694	1.32
ExpDB2 and ContDB2	Each ear separately	0.851	0.637	1.39

For the either-ear case, the *d'* values are somewhat higher than for the rM-NIHL method with the original criteria or the revised criteria, although the differences were not significant. For the “either ear” case the lower and upper CIs were 1.12 and 1.95. The M-NIHL60+ method gave a reasonable balance between sensitivity and specificity, except that the specificity was only 0.581 based on ContDB2. Overall, it appears that the M-NIHL60+ method is preferable to the rM-NIHL method with the original criteria and perhaps to the rM-NIHL method with the revised criteria.

To assess whether the weighting of the different frequencies used in the M-NIHL60+ method was beneficial, a version of the method was evaluated in which the HTLs for all frequencies from 1 to 8 kHz were weighted equally. This “equal-weight” version gave only slightly worse performance than the version with different weights across frequencies: for the either-ear case, the number of “hits” for ExpDB1 was unchanged and the number of “false positives” for ContDB1 increased from 23 to 25, corresponding to a decrease in specificity from 0.623 to 0.590. Although the benefit of the weighting was small, it is theoretically appropriate to use it.

## Discussion

This paper has considered how pure-tone thresholds measured at a specific age can be used to diagnose M-NIHL. Of course, if a series of reliable audiograms is available, M-NIHL can also be diagnosed from the changes in the audiogram over time. However, in some countries, audiograms collected prior to, during, or at the end of military service are not obtained by qualified audiologists, and this can lead to high variability and systematic errors (Moore et al., 2026). Hence, it may be preferable to base diagnosis on a single audiogram obtained according to the recommended method of the British Society of Audiology (2018) or a similar method.

It is also important to note that factors other than the audiogram should be taken into account when making a diagnosis for an individual. Specifically, a full medical history should be obtained to rule out possible causes of hearing loss other than noise exposure (Moore et al., 2022a) and to take into account the effect of possible other causes (e.g. conductive loss may protect against NIHL). The types and durations of noise exposures of the individual should be documented, and it should be assessed how often the individual experienced a dulling of hearing or tinnitus following the exposures (Brungart et al., 2019, 2025), and to assess whether other symptoms associated with noise exposure are present, such as tinnitus and hyperacusis (Lowe & Moore, 2021). A final diagnosis of NIHL for a particular individual should take account of these aspects as well as the outcome of diagnostic tests such as the rM-NIHL or M-NIHL60+ methods.

This study showed that the rM-MIHL method, which was originally developed to diagnose M-NIHL for men aged up to 60 years, had reasonably high sensitivity (0.915) for the either-ear case when applied to a population of military noise-exposed individuals aged 61 to 80 years, but had poor specificity (0.475). By increasing the values of the criteria, Mag(Notch) and Mag(ExcessHF), it was possible to obtain a better balance between sensitivity and specificity, but the overall diagnostic performance, based on the values of *d'* for the either-ear case, was only moderate (*d'* = 1.27). A new diagnostic method, M-NIHL60+, based on a weighted average across frequency of threshold elevations relative to AAHL values, also gave a reasonable balance between sensitivity and specificity and gave somewhat (but not significantly) higher *d'* values than for the rM-NIHL method with revised criteria (for the either-ear case, *d'* was 1.48 based on ExpDB1 and ContDB1, and 1.54 based on ExpDB2 and ContDB2). The M-NIHL60+ method gave significantly higher *d*′ values than the CLB method. Therefore, the M-NIHL60+ method is recommended for the diagnosis of M-NIHL for men aged 61-80 years.

For 61- to 80-year-old men, the overall diagnostic performance of the original rM-NIHL method, the rM-NIHL method with revised criteria, and the M-NIHL60+ method was lower than found for the rM-NIHL method applied to men aged up to 60 years (*d*′ was 2.37 for this age group). This is as expected, since with increasing age AAHL increases and tends to “swamp” the effects of noise exposure early in life.

Comparison of the audiograms for noise-exposed military personnel in younger groups (60 years old or below, taken from previous studies) and older groups (61-80 years old) revealed two aspects consistent with the “acceleration-deceleration” hypothesis, namely that exposure to noise during military service can accelerate the subsequent progression of hearing loss with increasing age for frequencies where hearing loss at the end of military service is mild or absent (typically low frequencies), but has no effect or slows the subsequent progression of hearing loss for frequencies where the hearing loss is moderate or severe. These aspects are:

(1) For younger noise-exposed individuals hearing loss is on average markedly greater for the left than for the right ear, especially for frequencies of 4, 6, and 8 kHz. This asymmetry across ears is small for older noise-exposed individuals and is similar to that for control non-exposed individuals, suggesting that the increase in hearing loss with increasing age occurs at a slower rate for frequencies where the hearing loss is relatively large early in life than for frequencies where the hearing loss early in life is mild.
(2) The age-corrected thresholds (HTLs relative to AAHLs) for younger noise-exposed men are markedly greater at high frequencies (4 and 6 kHz) than at l kHz. In contrast, the age-corrected thresholds for noise-exposed older men vary less across frequency, consistent with the idea that age-related deterioration in HTLs occurs at a higher rate for frequencies where the noise-exposure initially had little effect than for frequencies where the noise exposure initially had a large effect.

It should be noted that the differential effects of frequency described above do not directly indicate whether acceleration or deceleration is occurring; they merely indicate different age-related changes in HTL depending on the initial HTL. Also, the effects may partly reflect differences between longitudinal data, the “gold standard” for assessing changes in hearing with age (Brant & Fozard, 1990; Dillard et al., 2024, 2025c), and cross-sectional data, as presented here. Brant and Fozard (1990) noted that for a population sample the rate of change of HTL with increasing age for older males was faster for frequencies from 0.5 to 2 kHz than for higher frequencies, “since their hearing has already diminished at the high frequencies”. Similarly, Dillard et al. (2024) found that participants aged above 70 years had a greater rate of threshold change than younger participants for frequencies from 0.25 to 1 kHz, but the rate of change at higher frequencies was similar for those aged over 70 years and those aged 60-69 years.

As noted above, the older exposed group tested here showed less asymmetry across ears than for military personnel aged less than 60 years, as assessed in other studies. The marked asymmetry for younger men probably occurs because noise exposure during military service, and resultant effects on hearing, are usually asymmetrical (but to varying extents and sometimes with more exposure and loss for the right ear than for the left) (Lowe & Moore, 2021). For some of the older men tested here, the diagnostic criteria using the rM-NIHL method with revised criteria and/or the M-NIHL60+ method were met for one ear only. This does not imply that there was no NIHL for the ear that did not meet the diagnostic criteria. Rather, if one ear meets the diagnostic criteria, then any hearing loss greater than that expected from age is probably a result of noise damage for both ears. This principle should of course extend to those aged less than 60 years of age who are exposed to noise during military service.

## Strengths and Limitations

One strength of this paper is that it used well-screened, reasonably large samples of military-noise-exposed and non-noise-exposed older men, for whom audiometric threshold measures obtained to a high standard were available. A second strength is that the paper provides a new method for diagnosing M-NIHL for older men with higher diagnostic accuracy than the CLB method and slightly but not significantly higher diagnostic accuracy than the rM-NIHL method with revised criteria. A third strength is that the paper addresses the important public health question about whether military noise exposure that typically occurs in younger adults changes the progression of hearing loss later in life.

One limitation of this study is that the noise-exposed groups were not random or representative samples of former military personnel. The samples were limited to those claiming compensation for NIHL and/or tinnitus and/or hyperacusis (regardless of whether their claims were successful). This increased the likelihood of them having M-NIHL, but is associated with the risk that the hearing loss was exaggerated. This risk is mitigated by the fact that where faking is suspected, audiometric thresholds can be assessed objectively using cortical evoked-response audiometry (CERA). This was done for some of the individuals in the exposed databases, and in all cases the CERA results agreed well with the audiometric thresholds measured behaviorally.

Another limitation is that the noise-exposed and control groups were not matched precisely in age and were not matched in terms of some factors that can slightly affect HTLs, such as alcohol consumption, smoking, socio-economic status, and educational level (Davis, 1995; Dawes et al., 2014; Dillard et al., 2026). Also, the noise-exposed groups were probably less highly screened than the control groups in terms of exposure to leisure noise or exposure to noise following military service. However, it seems likely that noise exposure during military service far outweighed exposure from leisure activities or jobs outside military service (Jokel et al., 2019). Hence, M-NIHL for the noise-exposed groups, when present, was probably caused mainly by noise exposure during military service.

## Summary and Conclusions

When applied to men aged 61 to 80 years, for the either-ear case the rM-NIHL diagnostic method had high sensitivity but poor specificity, and a moderate overall diagnostic performance (*d*′ = 1.31). The CLB method had lower overall diagnostic performance (*d*′ = 0.826), although with slight overlap of the 95% CIs. Modifying the rM-NIHL method by increasing the criterion values of Mag(Notch) and Excess(HF) led to a better balance between sensitivity and specificity, but overall diagnostic performance remained moderate.

A new diagnostic method, denoted M-NIHL60+, based on a weighted average of the age-corrected hearing thresholds across all frequencies from 1 to 8 kHz, gave a reasonable balance between sensitivity and specificity and gave significantly higher diagnostic performance than the CLB method and somewhat (but not significantly) higher diagnostic performance (*d*′ = 1.54) than the rM-NIHL method or the rM-NIHL method with revised criteria. Hence, the M-NIHL60+ method is recommended for the diagnosis of M-NIHL for men aged 61 to 80 years.

Comparison of the audiograms for noise-exposed individuals in younger groups and older groups, and comparison to non-exposed age-matched groups, revealed patterns consistent with the “acceleration-deceleration” hypothesis.

## Data Availability

The databases ExpDB1, ContDB1, ExpDB2 and ContDB2 are available from the first author on reasonable request.*

## References

[bibr1-23312165261466799] American Speech-Language-Hearing Association . (2005). Guidelines for manual pure-tone threshold audiometry. https://www.asha.org/policy/gl2005-00014 (Last accessed May, 2026.

[bibr2-23312165261466799] Brant L. J. Fozard J. L. (1990). Age changes in pure-tone hearing thresholds in a longitudinal study of normal human aging. The Journal of the Acoustical Society of America, 88(2), 813–820. 10.1121/1.399731 2212307

[bibr3-23312165261466799] British Society of Audiology . (2018). Recommended procedure: Pure-tone air-conduction and bone-conduction threshold audiometry with and without masking: Author.

[bibr4-23312165261466799] Brungart D. S. Barrett M. E. Schurman J. Sheffield B. Ramos L. Martorana R. Galloza H. (2019). Relationship between subjective reports of temporary threshold shift and the prevalence of hearing problems in military personnel. Trends in Hearing, 23, 2331216519872601. 10.1177/2331216519872601 31524086 PMC6747866

[bibr5-23312165261466799] Brungart D. S. Ellis G. M. Davidson A. Galloza H. Sheffield B. Schurman J. (2025). Not-so-normal hearing: Temporary hearing changes lead to chronic difficulties for listeners with ”normal. audiometric thresholds, 458, 109183. Hearing Research. 10.1016/j.heares.2025.109183 39864272

[bibr6-23312165261466799] Coles R. R. Lutman M. E. Buffin J. T. (2000). Guidelines on the diagnosis of noise-induced hearing loss for medicolegal purposes. Clinical Otolaryngology, 25(4), 264–273. 10.1046/j.1365-2273.2000.00368.x 10971532

[bibr7-23312165261466799] Davis A. (1995). Hearing in Adults: Whurr.

[bibr8-23312165261466799] Dawes P. Cruickshanks K. J. Moore D. R. Edmondson-Jones M. McCormack A. Fortnum H. Munro K. J. (2014). Cigarette smoking, passive smoking, alcohol consumption, and hearing loss. Journal of the Association for Research in Otolaryngology, 15(4), 663–674. 10.1007/s10162-014-0461-0 24899378 PMC4141428

[bibr9-23312165261466799] Dillard L. K. Humes L. E. Matthews L. J. Dubno J. R. (2025c). Noise exposure history and age-related changes to hearing. JAMA Otolaryngology Head and Neck Surgery, 151(3), 228–235. 10.1001/jamaoto.2024.4768 39786765 PMC11907306

[bibr10-23312165261466799] Dillard L. K. Matthews L. J. Bainbridge K. E. Johnson J. M. Dubno J. R. (2026). Smoking history and the rate of hearing decline in aging: Results from a longitudinal cohort study. Hearing Research, 469, 109486. 10.1016/j.heares.2025.109486 41319482 PMC13308747

[bibr11-23312165261466799] Dillard L. K. Matthews L. J. Dubno J. R. (2025a). Sex- and race-specific prevalence of hearing loss across the adult lifespan and associated factors. JAMA Otolaryngology Head and Neck Surgery, 151(6), 576–583. 10.1001/jamaoto.2025.0534 40310632 PMC12046519

[bibr12-23312165261466799] Dillard L. K. Matthews L. J. Dubno J. R. (2025b). Race- and sex-specific differences in the risk of incident hearing loss and associated factors. Scientific Reports, 15(1), 13524. 10.1038/s41598-025-96937-0 40253452 PMC12009290

[bibr13-23312165261466799] Dillard L. K. Matthews L. J. Maldonado L. Simpson A. N. Dubno J. R. (2024). Demographic factors impact the rate of hearing decline across the adult lifespan. Communications Medicine, 4(1), 171. 10.1038/s43856-024-00593-w 39215139 PMC11364848

[bibr14-23312165261466799] Gourevitch V. Galanter E. (1967). A significance test for one parameter isosensitivity functions. Psychometrika, 32(1), 25–33. 10.1007/BF02289402 5232570

[bibr15-23312165261466799] Green D. M. Swets J. A. (1974). Signal Detection Theory and Psychophysics: Krieger.

[bibr16-23312165261466799] Hederstierna C. Rosenhall U. (2016). Age-related hearing decline in individuals with and without occupational noise exposure. Noise Health, 18(80), 21–25. 10.4103/1463-1741.174375 26780958 PMC4918675

[bibr18-23312165261466799] ISO 7029 . (2024). Acoustics - Statistical distribution of hearing thresholds related to age and gender: International Organization for Standardization.

[bibr19-23312165261466799] ISO 8253-1 . (2010). Acoustics - Audiometric test methods - Part 1: Basic pure tone air and bone conduction threshold audiometry: International Organization for Standardization.

[bibr20-23312165261466799] Jokel C. Yankaskas K. Robinette M. B. (2019). Noise of military weapons, ground vehicles, planes and ships. The Journal of the Acoustical Society of America, 146(5), 3832–3838. 10.1121/1.5134069 31795677

[bibr21-23312165261466799] Keim R. J. (1969). Sensorineural hearing loss associated with firearms. Archives of Otolaryngology, 90(5), 581–584. 10.1001/archotol.1969.00770030583010 5347120

[bibr22-23312165261466799] Lowe D. Moore B. C. J. (2021). Audiometric assessment of hearing loss sustained during military service. The Journal of the Acoustical Society of America, 150(2), 1030–1043. 10.1121/10.0005846 34470327

[bibr24-23312165261466799] Moore B. C. J. (2020). Diagnosis and quantification of military noise-induced hearing loss. The Journal of the Acoustical Society of America, 148(2), 884–894. 10.1121/10.0001789 32873002

[bibr25-23312165261466799] Moore B. C. J. (2021). The effect of exposure to noise during military service on the subsequent progression of hearing loss. International Journal of Environmental Research and Public Health, 18(5), 2436. 10.3390/ijerph18052436 33801367 PMC7967570

[bibr26-23312165261466799] Moore B. C. J. Gockel H. E. (2026). Calculation of lower bounds on the sensitivity and specificity of diagnostic tests: relevance to the diagnosis of noise-induced hearing loss. International Journal of Audiology, (in press). 10.1080/14992027.2026.2636909 41766118

[bibr28-23312165261466799] Moore B. C. J. Lowe D. A. (2022). Does exposure to noise during military service affect the progression of hearing loss with increasing age? Trends in Hearing, 26, 1–11. 10.1177/23312165221076940 PMC8832625 35128984

[bibr30-23312165261466799] Moore B. C. J. Schlittenlacher J. (2023). Diagnosing noise-induced hearing loss sustained during military service using deep neural networks. Trends in Hearing, 27, 1–9. 10.1177/23312165231184982 PMC10408324 37550005

[bibr31-23312165261466799] Moore B. C. J. von Gablenz P. (2021). Sensitivity and specificity of a method for diagnosis of military noise-induced hearing loss. The Journal of the Acoustical Society of America, 149(1), 62–65. 10.1121/10.0002977 33514161

[bibr23-23312165261466799] Moore B. C. J. Cox G. Lowe D. A. Gockel H. E. (2026). On the accuracy and repeatability of occupational audiograms obtained by non-audiologists. Journal of Occupational and Environmental Medicine, 68(4), 305–308. 10.1097/JOM.0000000000003597 41181948

[bibr27-23312165261466799] Moore B. C. J. Humes L. E. Cox G. Lowe D. A. Gockel H. E. (2022b). Modification of a method for diagnosing noise-induced hearing loss sustained during military service. Trends in Hearing, 26, 1–9. 10.1177/23312165221145005 PMC9761234 36518073

[bibr29-23312165261466799] Moore B. C. J. Lowe D. A. Cox G. (2022a). Guidelines for diagnosing and quantifying noise-induced hearing loss. Trends in Hearing, 26, 1–21. 10.1177/23312165221093156 PMC9052822 35469496

[bibr32-23312165261466799] Niskar A. S. Kieszak S. M. Holmes A. E. Esteban E. Rubin C. Brody D. J. (2001). Estimated prevalence of noise-induced hearing threshold shifts among children 6 to 19 years of age: the Third National Health and Nutrition Examination Survey, 1988-1994, United States. Pediatrics, 108(1), 40–43. 10.1542/peds.108.1.40 11433052

[bibr33-23312165261466799] Passchier-Vermeer W. (1974). Hearing loss due to continuous exposure to steady-state broad-band noise. The Journal of the Acoustical Society of America, 56(5), 1585–1593. 10.1121/1.1903482 4427029

[bibr34-23312165261466799] Phillips S. L. Henrich V. C. Mace S. T. (2010). Prevalence of noise-induced hearing loss in student musicians. International Journal of Audiology, 49(4), 309–316. 10.3109/14992020903470809 20233141

[bibr35-23312165261466799] Pudrith C. Phillips S. Labban J. (2022). Association of self-reported noise exposure and audiograms processed with algorithms proposed to quantify noise-induced hearing loss. International Journal of Audiology, 61(10), 809–817. 10.1080/14992027.2021.1983216 34634215

[bibr36-23312165261466799] von Gablenz P. Holube I. (2016). Hearing threshold distribution and effect of screening in a population-based German sample. International Journal of Audiology, 55(2), 110–125. 10.3109/14992027.2015.1084054 26418731

